# Association between dietary folate intake and severe headache or migraine in adults: a cross-sectional study of the National Health and Nutrition Examination Survey

**DOI:** 10.3389/fnut.2024.1456502

**Published:** 2024-11-26

**Authors:** Dehua Zhao, Xiaoqing Long, Jisheng Wang

**Affiliations:** Department of Clinical Pharmacy, The Third Hospital of Mianyang, Sichuan Mental Health Center, Mianyang, Sichuan, China

**Keywords:** folate, severe headache or migraine, logistic regression, restricted cubic spline regression, cross-sectional study

## Abstract

**Background:**

The aim of this study was to assess the association between dietary folate intake and severe headache or migraine.

**Methods:**

This cross-sectional study utilized the National Health and Nutrition Examination Survey (NHANES) data from 1999 to 2004. Weighted logistic regression models, restricted cubic spline (RCS) regression, sensitivity analysis, and stratified analyses were performed to evaluate the association between dietary folate intake and severe headache or migraine.

**Results:**

A total of 4,107 participants were included, with 704 individuals (17.14%) experienced severe headache or migraine. After adjusting for all covariates, an independent association was found between dietary folate intake and severe headache or migraine (OR = 0.77, 95% CI: 0.64–0.93, *p* = 0.005). When folate intake was categorized, individuals in Q2 (251.21–356.00 μg/d), Q3 (356.19–514.00 μg/d), and Q4 (≥515.00 μg/d) had ORs of 0.95 (95% CI: 0.75–1.20, *p* = 0.660), 0.86 (95% CI: 0.67–1.12, *p* = 0.266), and 0.65 (95% CI: 0.48–0.89, *p* = 0.007), respectively, compared to those in Q1 (≤251.00 μg/d). The RCS regression showed a linear negative relationship between dietary folate intake and severe headache or migraine. Stratified and sensitivity analyses yielded similar results.

**Conclusion:**

There was a linear negative relationship between dietary folate intake and migraine.

## Introduction

Migraine is a prevalent chronic neurovascular disease that has a significant impact on global public health, affecting around 1.04 billion individuals globally (1). Migraine episodes often recur and are characterized by intense headaches, accompanied by symptoms of autonomic nervous system dysfunction like nausea, vomiting, photophobia, and phonophobia (2). In addition, Migraines can lead to various health complications, such as cerebral white matter lesions, cognitive decline, and cerebral infarction (3). Medical conditions such as anxiety, depression, and other emotional disorders greatly diminish the quality of life for patients (4). Recent studies have revealed a connection between nutrients and severe headache or migraine, such as niacin, zinc, fiber, potassium, sodium, vitamin B2, thiamine, and riboflavin (5–13). Some nutrients may either trigger severe headache or migraine attacks or reduce the prevalence of migraine (12, 13). Folate, a B vitamin essential for one-carbon metabolism and DNA methylation, has demonstrated potential in decreasing migraine frequency and symptoms in female patients with aura-type migraine (14). Few studies have assessed the relationship between dietary folate intake and severe headache or migraine prevalence in the general population. Therefore, we conducted a cross-sectional study based on the National Health and Nutrition Examination Survey (NHANES) to assess the association between dietary folate intake and severe headache or migraine in adults.

## Methods

### Study population

The present cross-sectional study was conducted using the NHANES data from 1999 to 2004. We selected these cycles because these were the only cycles including severe headache or migraine questionnaires. Questionnaire data on severe headache or migraine is not available in other NHANES cycles. Our study included participants aged 20 and above who completed the survey. Pregnant females and participants with missing data on severe headache or migraine, dietary folate intake, or covariates were excluded from the analysis. Since all individuals had already signed an informed consent form prior to participating in the NHANES, no additional Institutional Review Board approval was needed for this secondary analysis.

### Dietary folate intake

Data on dietary folate intake were collected through the 24 h interview questionnaires during the investigation. The dietary intake data were utilized to estimate the types and quantities of foods and beverages consumed during the 24 h period before the interview, as well as to assess the intakes of energy, nutrients, and other food components derived from those foods and beverages. Dietary intake data between 1999 to 2001 were extracted using the NHANES computer-assisted dietary interview system, while data between 2002 to 2004 were extracted using the Automated Multiple Pass Method of the United States Department of Agriculture. Participants were classified into four groups based on their dietary folate intake.

### Migraine assessment

Participants were asked the question, “Have you experienced a severe headache or migraine in the past 3 months?” to determine if they had severe headache or migraine. Those who answered “yes” were classified as having severe headache or migraine.

### Covariates

Directed acyclic graphs (DAGs) are commonly used to visualize causal assumptions and guide the progressive inclusion of covariates in analyses *a priori* (15, 16). Therefore, we employed a DAG to depict the assumed relationships among the included variables (Supplementary Figure S1). Based on the results of DAG, we included several confounding factors as covariates, including age, gender, race/ethnicity, marital status, smoking status, drinking status, family income, education level, body mass index (BMI), C-reactive protein (CRP), physical activity, energy, proteins, carbohydrates, fat, diabetes, hypertension, hypercholesterolemia, stroke, and coronary heart disease.

Nutrient intake data including energy, proteins, carbohydrates, and fat were obtained through 24 h dietary recall interviews. Demographics data including age, gender, race/ethnicity, marital status, family income, and education level were obtained by a household interview. Family income was calculated by the poverty income ratio (PIR). Race/ethnicity was classified as Mexican American, Non-Hispanic White, Non-Hispanic Black, other Hispanic, and other race. The education level was categorized into three groups: less than high school, high school diploma, and more than high school. Marital status was classified as married, living with a partner, and living alone. BMI was calculated using a standardized technique based on weight and height. CRP was quantified by latex-enhanced nephelometry. Smoking status, drinking status, physical activity and comorbidities were retrieved from the NHANES questionnaire data file. The Smoking status includes an extensive array of questions on cigarette and tobacco use. Smoking status was defined as never smokers (less than 100 cigarettes), current smokers, and former smokers (over 100 cigarettes but quit). Information on alcohol intake was collected by personal interview in the mobile examination center (MEC). The questions in this section pertained to alcohol use over a lifetime and within the past 12 months for individuals aged 20 and older. According to previous studies, drinking status was classified into two groups: participants who consumed more than 12 alcoholic drinks per year and those who consumed fewer than 12 alcoholic drinks per year in their lifetime (10, 17). A drink refers to a 12 oz. beer, a 4 oz. glass of wine, or an ounce of liquor. Participants’ physical activity levels were determined based on their engagement in moderate and vigorous activities over the past 30 days. Moderate activities were described as those lasting at least 10 min and resulting in only light sweating or a slight to moderate increase in heart rate or breathing. Conversely, vigorous activities were defined as any activities in which participants experienced heavy sweating or significant elevations in heart rate or breathing for at least 10 min during the same 30-day period. The definition of comorbidities was based on the questionnaire of whether the doctor had been informed of the condition in the past.

### Statistical analysis

According to NHANES guidelines, sample weights must be recalculated when combining data from multiple cycles. In this study, we used dietary weights for the weighted analysis. The combined analyses of NHANES 1999–2000 and 2001–2002 data utilized a four-year dietary weight (WTDR4YR) set. The 2003–2004 data utilized the dietary day-one sample weight (WTDRD1). The sampling weights for the years 1999–2004 were determined as follows: weights for 1999–2002 were calculated as 2/3 of WTDR4YR, and otherwise 1/3 of WTDRD1. Descriptive analysis was performed for all participants. Continuous variables were described as mean ± standard deviation (SD) for normal distributions or median and interquartile range (IQR) for skewed distributions. Categorical variables were described as numbers and percentages. The chi-square test, one-way ANOVA, and Kruskal-Wallis test were used to examine categorical variables, normal distributions, and skewed distributions, respectively. OR and 95% confidence interval (CI) were calculated to explore the association between dietary folate intake and severe headache or migraine using weighted multivariable logistic regression. The basic characteristics of the study population showed that gender, race/ethnicity, education level, marital status, drinking status, smoking status, stroke, and hypertension differed among the four dietary folate intake groups (*p* < 0.05). Therefore, we selected those variables for stratified analysis. In addition, previous studies indicated that age significantly influences the relationship between dietary folate intake and severe headache or migraine (13, 14). Thus, we categorized age as <60 years or ≥ 60 years and used it as a stratified variable for analysis. Heterogeneity and interactions across subgroups were evaluated using logistic regression models and likelihood ratio test, respectively. We performed a sensitivity analysis to evaluate the robustness of the results, using multiple imputation to address missing covariate data. Additionally, we also assessed the dose–response relationship between folate intake and severe headache or migraine by restricted cubic spline (RCS) with four knots at the 5th, 35th, 65th, and 95th percentiles of dietary folate consumption after adjusting for all covariates (18). A two-sided *p*-value <0.05 was considered as statistically significant. Statistical analyses were performed using the statistical software package R, version 3.4.3, and Free Statistics software, version 1.8.

## Results

### Basic characteristics of the study population

**Figure 1:**
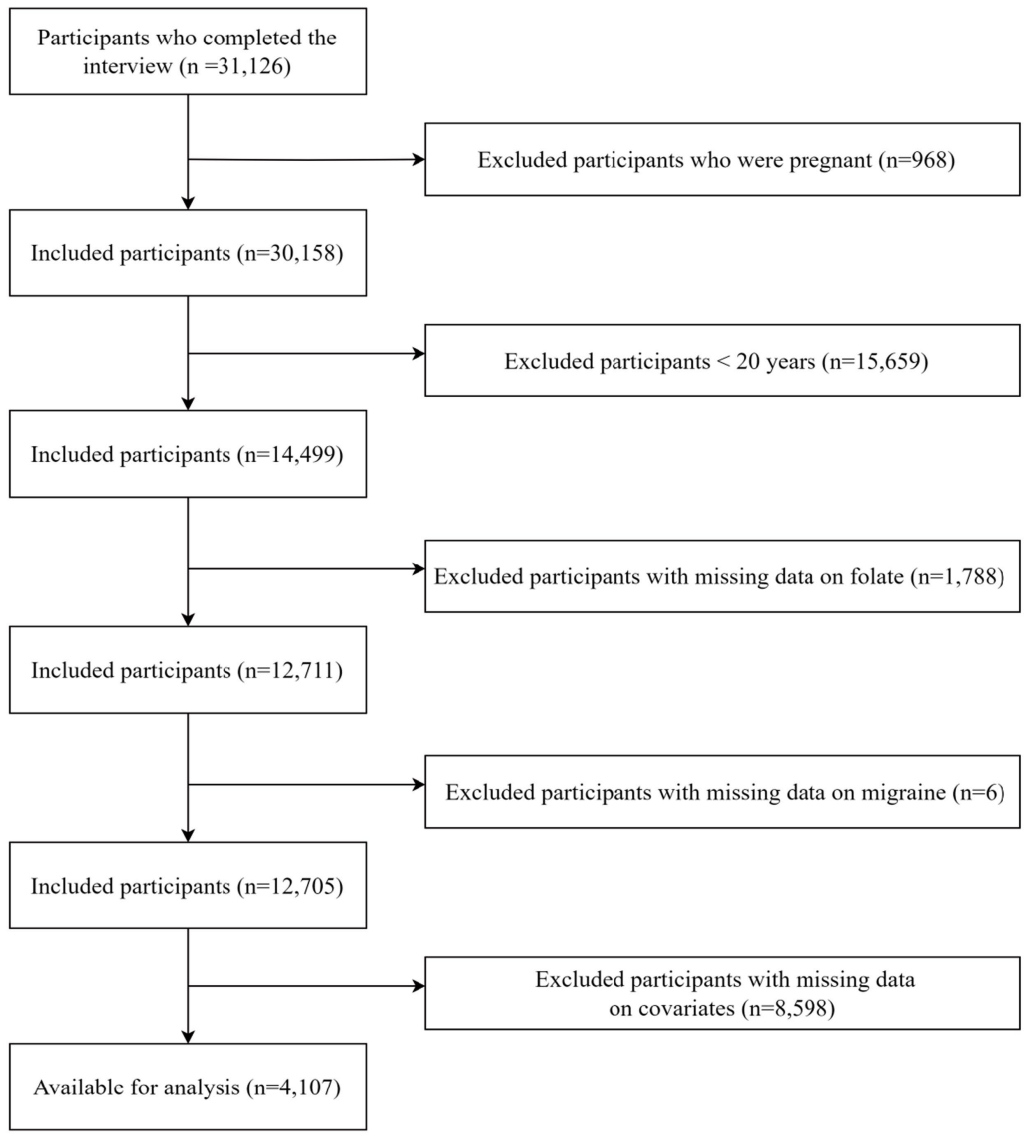
Flow chart of participants selection.

During the period of 1999 to 2004, a total of 31,126 participants were retrieved from the NHANES database. We excluded 968 pregnant participants, 15,659 participants under 20 years old, 1,788 participants with missing folate intake data, 6 participants with missing severe headache or migraine data, and 8,598 participants with missing covariate data. Finally, the remaining 4,107 participants were enrolled in our analysis. The process of participant exclusion was depicted in Figure 1.

Among the 4,107 participants, 704 individuals (17.14%) were found to have severe headache or migraine. The mean age of the participants was 52.96 ± 16.92, with 2,132 (51.91%) of them being male participants. The data indicated that dietary folate intake was generally higher in males, younger individuals, Non-Hispanic White individuals, participants who consumed more than 12 alcoholic drinks per year, former smokers, married individuals, and those with higher income and education levels. Additionally, higher dietary folate intake was associated with a lower incidence of hypertension, coronary heart disease, diabetes, stroke, and hypercholesterolaemia, as well as higher energy, protein, carbohydrate, and fat intake. The basic characteristics of included participants were listed in Table 1.

**Table 1:** Basic characteristics of the included participants.

Variables	Dietary folate intake (μg/d)				
	Total	Q1 (≤251.00)	Q2 (251.21–356.00)	Q3 (356.19–514.00)	Q4 (≥515.00)
No.	4,107	1,027	1,026	1,025	1,029
Gender, *n* (%)					
Male	2,132 (51.91)	391 (38.07)	488 (47.56)	570 (55.61)	683 (66.38)
Female	1975 (48.09)	636 (61.93)	538 (52.44)	455 (44.39)	346 (33.62)
Age (year), Mean ± SD	52.96 ± 16.92	53.52 ± 17.10	54.08 ± 17.26	53.74 ± 16.42	50.52 ± 16.66
Race/ethnicity, *n* (%)					
Mexican American	602 (14.66)	153 (14.9)	167 (16.28)	141 (13.76)	141 (13.7)
Other Hispanic	143 (3.48)	31 (3.02)	39 (3.8)	41 (4)	32 (3.11)
Non-Hispanic White	2,609 (63.53)	583 (56.77)	624 (60.82)	708 (69.07)	694 (67.44)
Non-Hispanic Black	608 (14.80)	221 (21.52)	163 (15.89)	101 (9.85)	123 (11.95)
Other race	145 (3.53)	39 (3.8)	33 (3.22)	34 (3.32)	39 (3.79)
Education level, *n* (%)					
Less than high school	713 (17.36)	219 (21.32)	217 (21.15)	146 (14.24)	131 (12.73)
High school diploma	906 (22.06)	254 (24.73)	228 (22.22)	220 (21.46)	204 (19.83)
More than high school	2,488 (60.58)	554 (53.94)	581 (56.63)	659 (64.29)	694 (67.44)
Marital status, *n* (%)					
Married	2,653 (64.60)	607 (59.1)	662 (64.52)	712 (69.46)	672 (65.31)
Living alone	1,280 (31.17)	373 (36.32)	316 (30.8)	277 (27.02)	314 (30.52)
Living with partner	174 (4.24)	47 (4.58)	48 (4.68)	36 (3.51)	43 (4.18)
Family PIR, Median (IQR)	3.40 (1.84, 5.00)	2.93 (1.48, 4.82)	3.16 (1.66, 5.00)	3.69 (2.15, 5.00)	3.96 (2.10, 5.00)
BMI (kg/m^2^), Mean ± SD	28.26 ± 5.93	28.96 ± 6.63	28.04 ± 5.55	28.28 ± 5.56	27.76 ± 5.87
CRP (mg/dl), Median (IQR)	0.21 (0.09, 0.45)	0.27 (0.11, 0.59)	0.21 (0.09, 0.46)	0.20 (0.09, 0.41)	0.16 (0.07, 0.34)
Diabetes, *n* (%)					
No	3,691 (89.87)	917 (89.29)	908 (88.5)	927 (90.44)	939 (91.25)
Yes	416 (10.13)	110 (10.71)	118 (11.5)	98 (9.56)	90 (8.75)
Severe headache or migraine, *n* (%)					
No	3,403 (82.86)	818 (79.65)	836 (81.48)	859 (83.8)	890 (86.49)
Yes	704 (17.14)	209 (20.35)	190 (18.52)	166 (16.2)	139 (13.51)
Physical activity, *n* (%)					
Moderate	3,440 (83.76)	854 (83.15)	858 (83.63)	871 (84.98)	857 (83.28)
Vigorous	667 (16.24)	173 (16.85)	168 (16.37)	154 (15.02)	172 (16.72)
Drinking status, *n* (%)					
< 12 alcohol drinks a year	1,123 (27.34)	343 (33.4)	282 (27.49)	253 (24.68)	245 (23.81)
≥ 12 alcohol drinks a year	2,984 (72.66)	684 (66.6)	744 (72.51)	772 (75.32)	784 (76.19)
Smoking status, *n* (%)					
Never smokers	2,159 (52.57)	529 (51.51)	544 (53.02)	519 (50.63)	567 (55.1)
Current smokers	628 (15.29)	203 (19.77)	165 (16.08)	130 (12.68)	130 (12.63)
Former smokers	1,320 (32.14)	295 (28.72)	317 (30.9)	376 (36.68)	332 (32.26)
Coronary heart disease, *n* (%)					
No	3,860 (93.99)	967 (94.16)	962 (93.76)	956 (93.27)	975 (94.75)
Yes	247 (6.01)	60 (5.84)	64 (6.24)	69 (6.73)	54 (5.25)
Stroke, *n* (%)					
No	3,981 (96.93)	985 (95.91)	992 (96.69)	993 (96.88)	1,011 (98.25)
Yes	126 (3.07)	42 (4.09)	34 (3.31)	32 (3.12)	18 (1.75)
Hypertension, *n* (%)					
No	2,636 (64.18)	604 (58.81)	649 (63.26)	667 (65.07)	716 (69.58)
Yes	1,471 (35.82)	423 (41.19)	377 (36.74)	358 (34.93)	313 (30.42)
Hypercholesterolaemia, *n* (%)					
No	2,473 (60.21)	628 (61.15)	599 (58.38)	604 (58.93)	642 (62.39)
Yes	1,634 (39.79)	399 (38.85)	427 (41.62)	421 (41.07)	387 (37.61)
Energy (kcal/day), Mean ± SD	2126.38 ± 995.52	1439.04 ± 569.98	1915.33 ± 656.33	2245.44 ± 742.70	2904.24 ± 1232.49
Protein (g/day), Median (IQR)	75.05 (54.39, 100.51)	54.38 (37.06, 72.90)	68.90 (52.83, 89.27)	79.62 (60.51, 101.32)	102.17 (76.94, 131.86)
Carbohydrate (g/day), Median (IQR)	239.85 (175.25, 321.14)	159.24 (112.94, 207.46)	215.12 (170.38, 270.14)	263.45 (215.72, 321.90)	339.04 (272.03, 439.63)
Fat (g/day), Median (IQR)	71.03 (48.42, 102.43)	52.74 (37.66, 70.12)	68.63 (48.71, 94.15)	79.33 (56.71, 107.19)	95.23 (64.75, 131.02)

SD, standard deviation; IQR, interquartile range; BMI, body mass index; CRP, C-reactive protein.

### Association between dietary folate intake and severe headache or migraine

**Figure 2:**
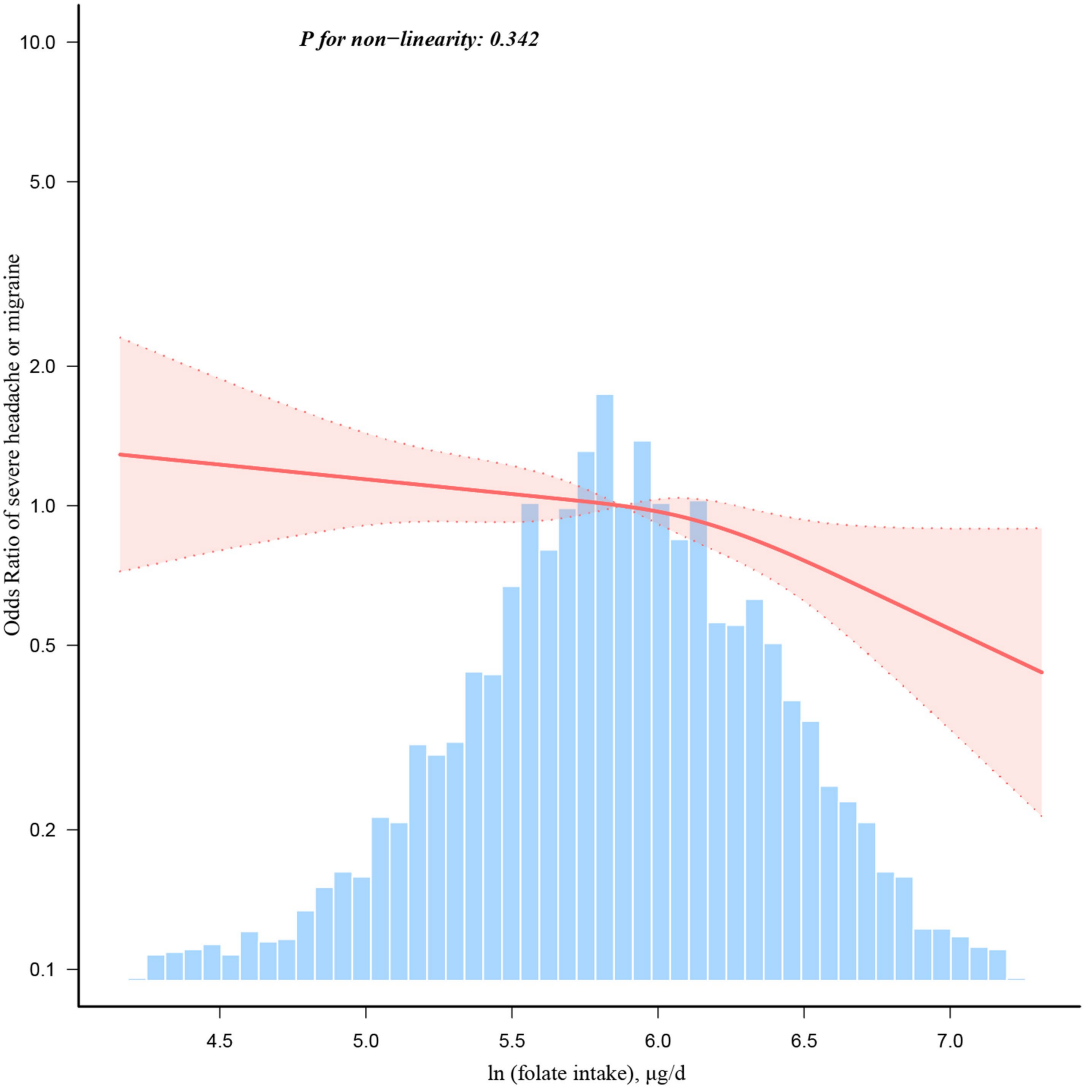
Dose–response relationship between dietary folate intake and severe headache or migraine. Adjustment factors included age, gender, race/ethnicity, marital status, education level, family income, smoking status, drinking status, BMI, physical activity, CRP, hypertension, hypercholesterolaemia, stroke, diabetes, coronary heart disease, energy, proteins, carbohydrates, and fat.

The severe headache or migraine was found to be associated with various factors, including gender, age, family income, BMI, physical activity, drinking status, and coronary heart disease, as evidenced by the univariate analysis in Table 2. In weighted multivariable logistic regressions (Table 3), dietary folate intake was investigated both as a continuous and categorical variable. After accounting for all covariates, an inverse association was observed between folate intake and severe headache or migraine when analyzed on a continuous scale (OR = 0.77, 95%CI: 0.64–0.93, *p* = 0.005). This association remained consistent when folate intake was categorized. Compared to individuals in the lowest folate intake category (Q1), those in Q2, Q3, and Q4 had ORs of 0.95 (95% CI: 0.75–1.20, *p* = 0.660), 0.86 (95% CI: 0.67–1.12, *p* = 0.266), and 0.65 (95% CI: 0.48–0.89, *p* = 0.007), respectively. The results were consistent across all models, demonstrating their strength and reliability. Moreover, based on the RCS analysis (Figure 2), there was a linear negative relationship between dietary folate intake and severe headache or migraine (*P* for non-linearity = 0.342).

**Table 2:** Association of covariates and severe headache or migraine.

Covariates	OR (95%CI)	*p*-value
Gender, *n* (%)		
Male	1 (Ref)	
Female	2.12 (1.79–2.51)	<0.001
Age (year)	0.97 (0.96–0.97)	<0.001
Race/ethnicity, *n* (%)		
Mexican American	1 (Ref)	
Other Hispanic	1.27 (0.82–1.95)	0.287
Non-Hispanic White	0.75 (0.59–0.94)	0.011
Non-Hispanic Black	0.96 (0.72–1.27)	0.762
Other race	1.06 (0.68–1.66)	0.803
Education level, *n* (%)		
Less than high school	1 (Ref)	
High school diploma	1 (0.77–1.29)	0.991
More than high school	0.88 (0.71–1.1)	0.259
Marital status, *n* (%)		
Married	1 (Ref)	
Living alone	1.09 (0.91–1.3)	0.337
Living with partner	1.62 (1.13–2.32)	0.009
Family PIR	0.87 (0.83–0.92)	<0.001
BMI (kg/m^2^)	1.02 (1.01–1.04)	<0.001
CRP (mg/dl)	1.07 (0.98–1.16)	0.135
Diabetes, *n* (%)		
No	1 (Ref)	
Yes	0.94 (0.71–1.23)	0.650
Physical activity, *n* (%)		
Moderate	1 (Ref)	
Vigorous	1.30 (1.05–1.6)	0.015
Drinking status, *n* (%)		
<12 alcohol drinks a year	1 (Ref)	
≥12 alcohol drinks a year	0.70 (0.59–0.83)	<0.001
Smoking status, *n* (%)		
Never smokers	1 (Ref)	
Current smokers	1.19 (0.96–1.48)	0.120
Former smokers	0.62 (0.51–0.75)	<0.001
Coronary heart disease, *n* (%)		
No	1 (Ref)	
Yes	0.53 (0.35–0.8)	0.003
Stroke, *n* (%)		
No	1 (Ref)	
Yes	1.14 (0.73–1.8)	0.564
Hypertension, *n* (%)		
No	1 (Ref)	
Yes	0.94 (0.79–1.12)	0.482
Hypercholesterolaemia, *n* (%)		
No	1 (Ref)	
Yes	1 (0.85–1.18)	0.994
Energy (kcal/day)	1.00 (1.00–1.00)	0.440
Protein (g/day)	1.00 (1.00–1.00)	0.118
Carbohydrate (g/day)	1.00 (1.00–1.00)	0.672
Fat (g/day)	1.00 (1.00–1.00)	0.821

CI, confidence interval; OR, odds ratio; BMI, body mass index; CRP, C-reactive protein.

**Table 3:** Association between dietary folate intake and severe headache or migraine.

Dietary folate intake (μg/d)	*N* (%)	Non-adjusted Model		Model 1		Model 2		Model 3	
		OR (95%CI)	*p*-value	OR (95%CI)	*p*-value	OR (95%CI)	*p*-value	OR (95%CI)	*p*-value
ln (folate)	704 (17.1)	0.72 (0.63–0.83)	<0.001	0.8 (0.7–0.93)	0.003	0.82 (0.71–0.95)	0.008	0.77 (0.64–0.93)	0.005
Quartiles									
Q1 (≤251.00)	209 (20.4)	1 (Ref)		1 (Ref)		1 (Ref)		1 (Ref)	
Q2 (251.21–356.00)	190 (18.5)	0.89 (0.71–1.11)	0.294	0.96 (0.76–1.20)	0.705	0.98 (0.78–1.24)	0.872	0.95 (0.75–1.20)	0.660
Q3 (356.19–514.00)	166 (16.2)	0.76 (0.60–0.95)	0.015	0.88 (0.7–1.12)	0.307	0.91 (0.72–1.16)	0.435	0.86 (0.67–1.12)	0.266
Q4 (≥515.00)	139 (13.5)	0.61 (0.48–0.77)	<0.001	0.70 (0.55–0.90)	0.005	0.72 (0.56–0.93)	0.011	0.65 (0.48–0.89)	0.007
Trend test			<0.001		0.005		0.011		0.009

CI, confidence interval; OR, odds ratio; ln (folate), folate intake values were natural logarithmised. Model 1 was adjusted for age, gender, race/ethnicity, marital status, education level, and family income. Model 2 was adjusted for age, gender, race/ethnicity, marital status, education level, family income, smoking status, drinking status, BMI, physical activity, CRP, hypertension, hypercholesterolaemia, stroke, diabetes, and coronary heart disease. Model 3 was fully adjusted, including age, gender, race/ethnicity, marital status, education level, family income, smoking status, drinking status, BMI, physical activity, CRP, hypertension, hypercholesterolaemia, stroke, diabetes, coronary heart disease, energy, proteins, carbohydrates, and fat.

### Stratified and sensitivity analyses

In gender, age, race/ethnicity, education level, marital status, drinking status, smoking status, stroke, and hypertension stratification, the effect was somewhat more pronounced in females, under 60 years old, Non-Hispanic White individuals, individuals with more than a high school education, those living alone, those consumed fewer than 12 alcoholic drinks per year, never smokers, those without stroke, and those without hypertension, but the difference between the effects in these groups was not statistically significant (Supplementary Figure S2).

The results remained robust in sensitivity analysis (Supplementary Table S1). After adjusting for all covariates, the dietary folate intake was inversely associated with severe headache or migraine (OR = 0.79, 95%CI: 0.72–0.86, *p* < 0.001). When folate intake was converted into a categorical variable, the adjusted ORs for Q2, Q3, and Q4 were 0.82 (95% CI: 0.72–0.93, *p* = 0.002), 0.82 (95% CI: 0.72–0.94, *p* = 0.005), and 0.69 (95% CI: 0.58–0.81, *p* < 0.001), respectively, compared to Q1.

## Discussion

Our cross-sectional study showed a significant negative association between dietary folate intake and severe headache or migraine. This association was consistently observed in subgroup analyses and RCS regression. These findings align with previous research, wherein it has been suggested that folate consumption could lower the incidence of severe headache or migraine (19–21). Moreover, a cohort study found that higher dietary folate intake was associated with lower severe headache or migraine frequency in women with severe headache or migraine (14). Furthermore, three clinical studies reported that pediatric patients with severe headache or migraine had lower levels of serum folic acid compared to pediatric patients without severe headache or migraine (22–24). Nevertheless, these studies focused on specific populations, such as female severe headache or migraine patients and pediatric severe headache or migraine patients, with relatively small sample sizes (24, 25). Therefore, we conducted a cross-sectional study using NHANES data to investigate the association between dietary folate intake and severe headache or migraine in the general population.

A linear negative relationship between dietary folate intake and severe headache or migraine was demonstrated in the RCS regression, which was consistent with the results of weighted multivariable logistic regression. In weighted multivariable logistic regressions, compared with individuals with the lowest folate intake Q1, the OR for severe headache or migraine was gradually decreased from Q2 to Q4, and this trend was statistically significant (*p* = 0.009). A dose–response relationship between folate intake and severe headache or severe headache or migraine was also found in a randomized, double-blinded, placebo-controlled trial (25). The results revealed that the dosage of 1 mg folic acid in combination with vitamin B6 and B12 was less effective in reducing severe headache or migraine-associated symptoms compared to the previously tested dosage of 2 mg folic acid in combination with vitamin B6 and B12 (25).

The subgroup analyses revealed that the OR for severe headache or migraine was greater than 1 in Mexican American individuals, individuals with less than a high school education, individuals living with a partner, and individuals with stroke. However, these associations were not statistically significant (*p* > 0.05), most likely due to the small sample sizes in these subgroups. On the other hand, in subgroups such as females, individuals aged less than 60 years, Non-Hispanic White individuals, individuals with a higher than high school education, married individuals, individuals living alone, non-drinkers, drinkers, never smokers, individuals without stroke, and individuals without hypertension, the OR for severe headache or migraine was found to be less than 1, indicating a statistically significant inverse association (*p* < 0.05). Therefore, consistent with the weighted multivariable logistic regression findings, subgroup analyses further support a reverse association between dietary folate intake and severe headache or migraine.

Multiple studies have shown that folate plays a significant role in the pathophysiology of severe headache or migraine (26–29). Folate is necessary for the metabolism of homocysteine to methionine (30). Homocysteine, a highly reactive amino acid, may be involved in the initiation and maintenance of severe headache or migraine by producing endothelial injury through impaired release of nitric oxide (31, 32). Based on this hypothesis, it has been suggested that folate can be used for severe headache or migraine prophylaxis by decreasing the levels of homocysteine. The mechanisms of severe headache or migraine pathogenesis remain unclear, but it is believed that folate plays a crucial role.

The NHANES database only reports the amount of each nutrient consumed by a participant daily, but lacks detailed calculations and information on specific food sources. As a result, data regarding the primary food sources of folate were not available in the NHANES database. Previous studies showed that folate-rich foods include legumes, leafy green vegetables, fruits, nuts, whole grains, and animal liver (27–30).

Our study has several strengths. First, the present study population in this research comprises a large, nationally representative sample of US adults, indicating sufficient study power and credible results. Second, we modified various potential confounders in order to mitigate their confounding impact. Third, we conducted various weighted multivariable logistic regression models to assess the relationship between dietary folate intake and severe headache or migraine. Results were consistent across all models, suggesting the robustness of the findings. Fourth, we conducted RCS regression to analyze the dose–response relationship between dietary folate intake and severe headache or migraine, offering practical recommendations. The present study also had some limitations. First, although we implemented weighted multivariable logistic regression models, RCS regression, and stratified analyses, we recognize that residual confounding effects from unmeasured or unknown factors may still exist. Second, the classification of participants with severe headache or migraine was based on a single question rather than the International Classification of Headache Disorders criteria. However, the prevalence of severe headache or migraine in our study (17.14%) was similar to the American migraine prevalence and prevention study (17.4%) (33), which supports the validity of our approach in identifying severe headache or migraine. Furthermore, several studies utilized NHANES data to assess the relationship between severe headache or migraine and nutrients, all employing the same definitions to define severe headache or migraine (5–13). Third, over the past two decades, changes in baseline participant information, dietary folate intake, and dietary folate fortification practices may have impacted the true relationship between dietary folate intake and severe headache or migraine. However, our findings were consistent with the recent studies (1, 14, 34). The randomized double-blind controlled trial demonstrated that folic acid was effective as an adjuvant in treating and preventing episodic severe headache or migraine (34). In addition, a cohort study demonstrated that folate intake was inversely associated with severe headache or migraine (14). Moreover, based on the systematic review and meta-analysis, serum folate levels in severe headache or migraine patients were lower than those of healthy controls, indicating that there was a relationship between folate and severe headache or migraine (1). In any case, our findings may prompt future controlled studies to verify the association between folate intake and severe headache or migraine. Fourth, the dietary data were primarily based on participants’ memory, which may introduce recall biases and potentially compromise the accuracy of the results. However, previous studies have shown that 24 h recalls provide more comprehensive information about the type and quantity of food consumed compared to food frequency surveys. Furthermore, the 24 h dietary recall interview used in the NHANES has undergone extensive evaluation (5–13). Fifth, Observational studies are subject to reverse causation, which makes the association between folate intake and severe headache or migraine somewhat ambiguous. It remains unclear whether severe headache or migraine result from reduced food intake or if severe headache or migraine lead to decreased food intake, as severe headache or migraine can decrease individuals’ appetite. Therefore, future studies are necessary to address this gap. Lastly, as our participants were exclusively from the United States of America, further studies are needed to confirm the generalizability of our findings to other populations.

## Conclusion

This study found a linear negative association between dietary folate intake and severe headache or migraine. These findings suggest that folate might be effective for preventing and treating severe headache or migraine.

## Data Availability

The original contributions presented in the study are included in the article/Supplementary material, further inquiries can be directed to the corresponding author.
