# Supplementary material for: Association between dietary folate intake and severe headache or migraine in adults: a cross-sectional study of the National Health and Nutrition Examination Survey
Source: Front Nutr. 2024 Nov 26;11:1456502. doi: 10.3389/fnut.2024.1456502 (PMC11629539; doi:10.3389/fnut.2024.1456502)
Supplement: Supplementary file 1 [file Table_1.docx]

**Supplementary Table 1** Results of sensitivity analysis

| Dietary folate intake (μg/d) | N (%) | Non-adjusted Model | | Adjusted Model | |
| --- | --- | --- | --- | --- | --- |
|  |  | OR (95%CI) | *P*-value | OR (95%CI) | *P*-value |
| ln (folate) | 2526 (19.9) | 0.78 (0.73-0.83) | <0.001 | 0.79 (0.72-0.86) | <0.001 |
| Quartiles |  |  |  |  |  |
| Q1 (<225.00) | 752 (23.7) | 1 (Ref) |  | 1 (Ref) |  |
| Q2 (225.00-333.87) | 627 (19.7) | 0.79 (0.70-0.89) | <0.001 | 0.82 (0.72-0.93) | 0.002 |
| Q3 (334.00.-481.57) | 608 (19.2) | 0.76 (0.68-0.86) | <0.001 | 0.82 (0.72-0.94) | 0.005 |
| Q4 (≥482.00) | 539 (16.9) | 0.66 (0.58-0.74) | <0.001 | 0.69 (0.58-0.81) | <0.001 |
| Trend test |  |  | <0.001 |  | <0.001 |

Abbreviations: CI, confidence interval; OR, odds ratio; ln (folate), folate intake values were natural logarithmised. The model adjusted for age, gender, race/ethnicity, marital status, education level, family income, smoking status, drinking status, BMI, physical activity, CRP, hypertension, hypercholesterolaemia, stroke, diabetes, coronary heart disease, energy, proteins, carbohydrates, and fat.
