# Supplementary material for: Association between dietary folate intake and severe headache or migraine in adults: a cross-sectional study of the National Health and Nutrition Examination Survey
Source: Front Nutr. 2024 Nov 26;11:1456502. doi: 10.3389/fnut.2024.1456502 (PMC11629539; doi:10.3389/fnut.2024.1456502)
Supplement: Supplementary file 2 [file Image_1.pdf]

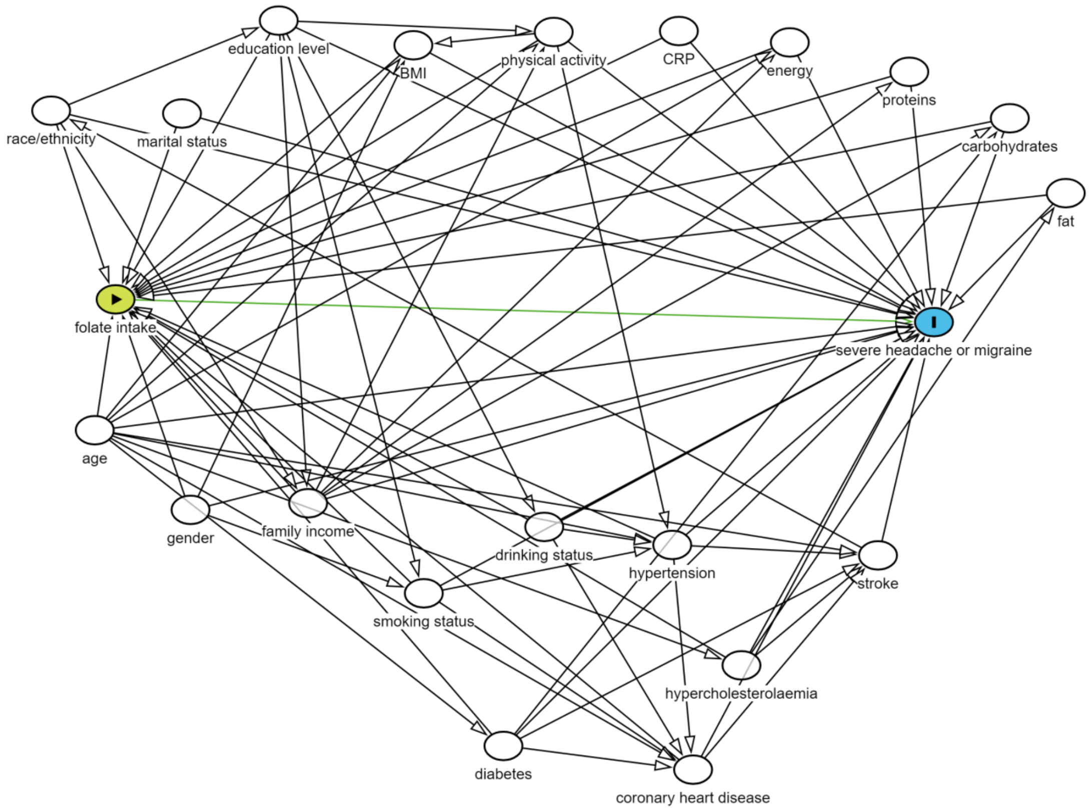

Supplementary figure 1 Directed acyclic graph depicting the assumed relationships between the included variables.

Subgroup	Event (%)	OR (95%CI)	P for interaction
**Gender**			**0.316**
Male	258 (12.10)	0.90 (0.66~1.24)	
Female	446 (22.58)	0.71 (0.56~0.91)	
**Age (year)**			**0.343**
<60	566 (22.49)	0.76 (0.62~0.94)	
≥60	138 (8.68)	0.77 (0.49~1.21)	
**Race/ethnicity**			**0.936**
Mexican American	119 (19.77)	1.11 (0.65~1.89)	
Other Hispanic	34 (23.78)	0.56 (0.11~2.91)	
Non−Hispanic white	405 (15.52)	0.72 (0.56~0.92)	
Non−Hispanic black	116 (19.08)	0.87 (0.60~1.28)	
Other race	30 (20.69)	0.66 (0.26~1.69)	
**Education level**			**0.729**
Less than high school	130 (18.23)	1.18 (0.70~1.97)	
High school diploma	165 (18.21)	0.71 (0.49~1.03)	
More than high school	409 (16.44)	0.73 (0.57~0.93)	
**Marital status**			**0.996**
Married	436 (16.43)	0.78 (0.61~0.99)	
Living alone	226 (17.66)	0.71 (0.52~0.97)	
Living with partner	42 (24.14)	1.18 (0.44~3.15)	
**Drinking status**			**0.658**
< 12 alcohol drinks a year	236 (21.02)	0.69 (0.48~0.97)	
≥ 12 alcohol drinks a year	468 (15.68)	0.78 (0.62~0.98)	
**Smoking status**			**0.234**
Never smokers	404 (18.71)	0.72 (0.56~0.92)	
Current smokers	135 (21.50)	0.73 (0.46~1.18)	
Former smokers	165 (12.50)	0.92 (0.61~1.40)	
**Stroke**			**0.477**
No	680 (17.08)	0.77 (0.63~0.92)	
Yes	24 (19.05)	1.50 (0.33~6.92)	
**Hypertension**			**0.350**
No	460 (17.45)	0.76 (0.60~0.96)	
Yes	244 (16.59)	0.80 (0.58~1.10)	

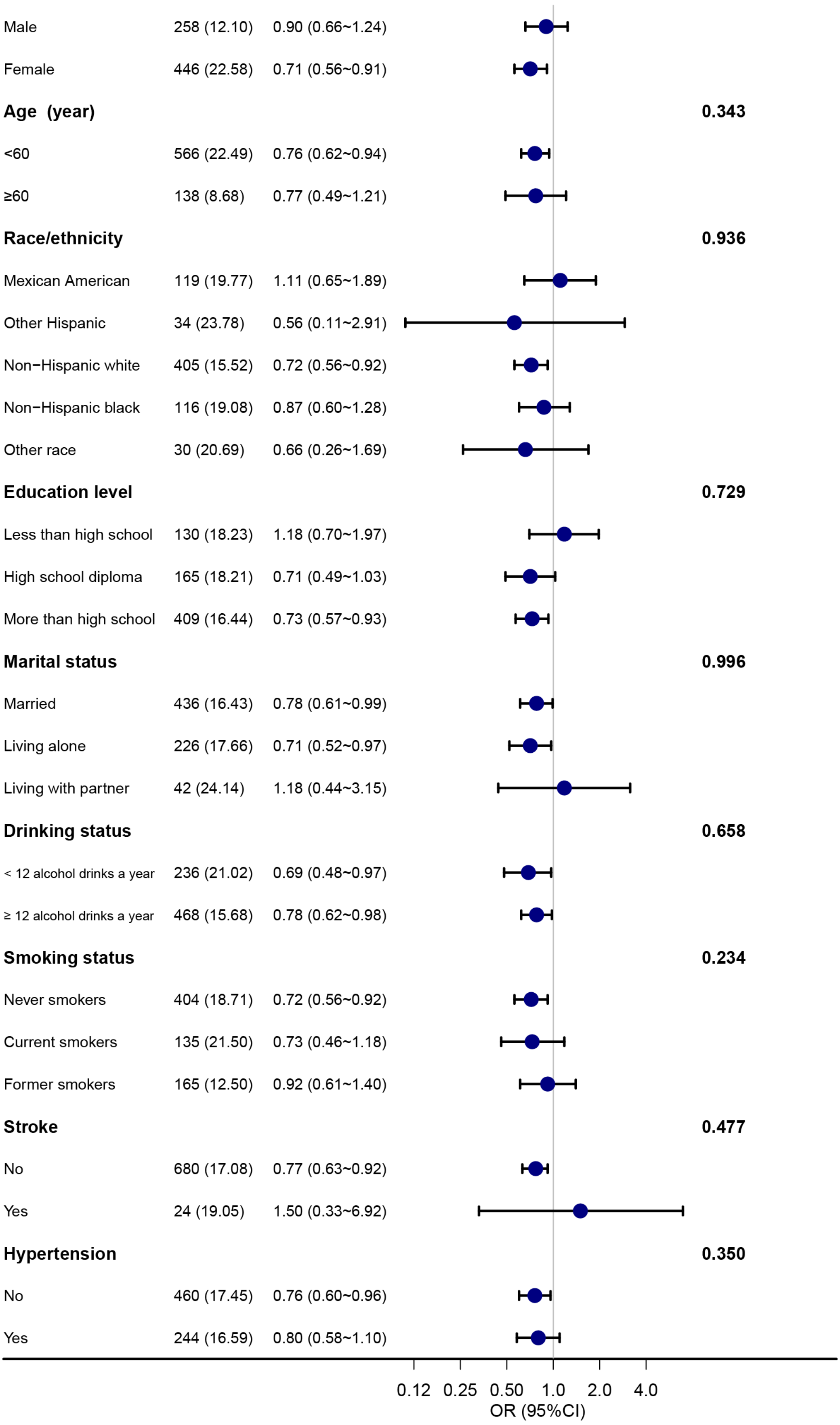

Supplementary Figure 2 Stratified analyses of the association between dietary folate intake and severe headache or migraine according to baseline characteristics. Except for the stratification component itself, the stratifications were adjusted for age, gender, race/ethnicity, marital status, education level, family income, smoking status, drinking status, BMI, physical activity, CRP, hypertension, hypercholesterolaemia, stroke, diabetes, coronary heart disease, energy, proteins, carbohydrates, and fat.
